# Gut microbiota display alternative profiles in patients with early-onset colorectal cancer

**DOI:** 10.3389/fcimb.2022.1036946

**Published:** 2022-10-27

**Authors:** Huan Xiong, Jiaqi Wang, Zewen Chang, Hanqing Hu, Ziming Yuan, Yihao Zhu, Zhiqiao Hu, Chunlin Wang, Yunxiao Liu, Yang Wang, Guiyu Wang, Qingchao Tang

**Affiliations:** ^1^ Department of Colorectal Surgery, the Second Affiliated Hospital of Harbin Medical University, Harbin, China; ^2^ Department of Urology Surgery, National Cancer Center, Chinese Academy of Medical Sciences, Peking Union Medical College Cancer Hospital Surgery, Beijing, China

**Keywords:** gut microbiota, colorectal cancer, early onset, 16S rRNA, functional annotation

## Abstract

**Background:**

The incidence of early-onset colorectal cancer (EOCRC) is increasing worldwide. This study aimed to explore whether there is an alternative gut microbiota profile in patients with early-onset colorectal cancer.

**Methods:**

A total of 24 patients with EOCRC, 43 patients with late-onset colorectal cancer and 31 young volunteers were included in this study. The diversity of their fecal bacteria was explored using 16S ribosomal RNA gene sequencing. Cluster of ortholog genes (COG) functional annotation and Kyoto encyclopedia of genes and genomes (KEGG) were used to detect enrichment pathways among the three groups.

**Results:**

Community separations were observed among the three groups. The Shannon index of the EOCRC group was significantly lower than the LOCRC group (P=0.007) and the NC group (P=0.008). Both PCoA analysis (Principal co-ordinates analysis, P=0.001) and NMDS (non-metric multidimensional scaling, stress=0.167, P=0.001) analysis indicated significant difference in beta diversity among the three groups. *Fusobacteria*, *Bacteroidetes*, and *Clostridia* were the most abundant bacteria in the EOCRC group, LOCRC group, and NC group, respectively. The results of COG showed that transcription (P=0.01398), defense mechanisms (P=0.04304), inorganic ion transport and metabolism (P=0.00225) and cell wall/membrane/envelope biogenesis (P=0.02534) were differentially expressed among the three groups. The KEGG modules involved in membrane transport (P=0.00856) and porphyrin and chlorophyll metabolism (P=0.04909) were differentially expressed among the three groups.

**Conclusion:**

Early-onset colorectal cancer patients have a different gastrointestinal microbiota derangement compared to late-onset colorectal cancer patients. This dysbiosis can be reflected in the species diversity of the microbiota, the abundance of bacteria, and the abnormal functional predictions.

## Introduction

Colorectal cancer is the third most common cancer in terms of incidence and second in terms of cancer-related mortality worldwide (Sung et al., 2021). Approximately ten percent of all patients initially diagnosed with colorectal cancer are younger than 50 years of age (Collaborative et al., 2021). Early-onset colorectal cancer (EOCRC) is generally defined as colorectal cancer diagnosed before the age of 50 years (Patel et al., 2022). The incidence of late-onset colorectal cancer has declined due to preventive screening recommendations over the past 10 years (Araghi et al., 2019; Siegel et al., 2020; Sinicrope, 2022). However, the incidence and cancer-related mortality of EOCRC have increased significantly and will continue to show an increasing trend over in next 10 years (Bailey et al., 2015; Araghi et al., 2019; Collaborative et al., 2021).

EOCRC always displays adverse clinical and histopathological features, yet the causes are unclear (Chang et al., 2012; Kneuertz et al., 2015; Saraste et al., 2020). In addition to the inherent genetic factors such as family history and germline gene mutations, poor dietary habits, smoking, alcohol, and antibiotics were considered risk factors for EOCRC (Chang et al., 2021; Patel et al., 2022). These risk factors can interact with the gut microbiota (Song and Chan, 2019), and their effects on the host can all be directly reflected by changes in the structure and abundance of the gut microbiota.

The gut microbiota, as an ecosystem in direct contact with the gut mucosa, is the potential cause of colorectal cancer (Garrett, 2019). Alterations in the structure of the intestinal microbiota can contribute to the development and progression of intestinal diseases. Increased abundance of certain specific microorganisms (*Fusobacterium nucleatum*, *Prevotella intermedia*, *Bacteroides fragilis*, *Porphyromonas asaccharolytica*, etc.) can increase the risk of colorectal carcinogenesis through inflammatory responses, evasion of tumor immune responses, and activation of pre-tumor signaling pathways (e.g., β-catenin) (Hernandez-Luna et al., 2019; Wong and Yu, 2019). However, probiotics such as *Lactobacillus* and *Streptococcus thermophilus* were significantly less abundant in the gut of colorectal cancer patients (Li et al., 2021). Most of the current data used to explore the microbiota structure of patients with colorectal cancer are derived from late-onset colorectal cancer (Murphy et al., 2019), with few studies characterizing the gut microbiota in early-onset colorectal cancer. In this study, we propose to use high-throughput DNA sequencing technology to analyze the gut microbiota of early onset colorectal cancer patients from our center and to conduct a preliminary.

## Material and methods

### Sample collection

The fecal specimens of all patients in this study were obtained from the Department of Colorectal Surgery, Second Affiliated Hospital of Harbin Medical University from July 2018 to June 2020. The inclusion criteria for this study were: 1) Patients with colorectal cancer diagnosed with histopathology, and healthy young volunteers without tumors by gastroscopy; 2) Consent for us to collect their feces. The exclusion criteria were: 1) Had taken antibiotics, probiotics, corticosteroids or received fecal microbiota transplantation treatment within 3 months prior to sample collection; 2) Had a familial history of colorectal cancer; 3) Had used evacuant or undergone colonoscopy within 1 week prior to sample collection; 4) Had undergone abdominal surgery or other invasive treatment within 3 months prior to sample collection; 5) Had been diagnosed with multiple primary cancers; 6) Had a history of other cancer or inflammatory bowel disease; 7) Contamination of specimens as a result of failure to collect according to prescribed protocols (Di Segni et al., 2018); 8) Incomplete clinical information. The recruited sporadic CRC patients were divided into two groups based on age: the EOCRC group, aged < 50 years; LOCRC group, aged ≥ 55 years. All recruited young healthy volunteers were less than 50 years of age and they were included in the NC group. Clinical and pathological characteristics of CRC patients including age, gender, body mass index (BMI), history of drinking, tumor location, histological classification of tumors, and TNM stage were collected. The collected information of healthy volunteers included age, gender, BMI, and history of drinking. The stools were rapidly frozen in liquid nitrogen for 30 seconds after acquisition and stored at -80°C until DNA was extracted.

### 16S ribosomal RNA gene sequencing

Microbial DNA was extracted from fecal samples using the E.Z.N.A. @ Soil DNA Kit (Omega Bio-tek, Norcross, GA, U.S.) according to the manufacturer’s protocol. The specific steps were performed according to the instructions. Final DNA concentration and purification were determined by NanoDrop 2000 UVVisspetrophotometer (Thermo Scientific, Wilmington, USA), and DNA quality was checked by 1% agarose gel electrophoresis. The extracted DNA was stored in a refrigerator at -80°C. The V3-V4 hypervariable regions (the 338F ~ 806R regions) of the bacterial 16S rRNA gene were amplified by high-throughput sequencing on a thermal cycler PCR system (GeneAmp 9700, ABI, USA) with primer sequences: 338F: 5’-ACTCCTACGGGAGGCAGCAG-3’, 806R: 5 ‘-GGACTACHVGGGTWTCTAAT-3’. The amplified DNA was further purified using the AxyPrep DNA Gel Extraction Kit (Axygen Biosciences, Union City, CA, USA) and quantified using QuantiFluor™-ST (Promega, USA) according to manufacturer’s established guidelines. Then, the normalized equimolar concentrations of each amplicon were pooled and sequenced on the Illumina MiSeq platform (Illumina, San Diego, USA) using 2 × 300 bp chemistry according to the standard protocol from Majorbio bio Pharm Technology Co. (Shanghai, China).

## Processing of sequencing data

The raw fastq files were filtering and trimming using Trimmomatic and merged by FLASH with the following criteria: (i) The reads were truncated at any site receiving an average quality score <20 over a 50 bp sliding window. (ii) Sequences whose overlap being longer than 10 bp were merged according to their overlap with mismatch no more than 2 bp. (iii)Sequences of each sample were separated according to barcodes (exactly matching) and Primers (allowing 2 nucleotide mismatching), and reads containing ambiguous bases were removed. Operational taxonomic units (OTUs) were calculated *via* clustering by average neighbor principle at 97% genetic similarity using UPARSE (version 7.1 http://drive5.com/uparse/). The chimeric sequences were identified and deleted after the comparison of the identified taxa. The classification of each 16S rRNA gene sequence was analyzed against the Silva (SSU123) 16S rRNA database using the RDP classifier algorithm (http://rdp.cme.msu.edu/) with a 70% confidence level (threshold).

### Analysis of processed sequencing data

Alpha diversity between the three groups was compared using Shannon index, Simpson index and the Simpson index. Beta diversity comparison between the three groups was done by PCoA analysis (Principal co-ordinates analysis), NMDS (Non-metric multidimensional scale) analysis and PLS-DA analysis (partial least squares discriminant analysis). PCoA analysis and NMDS analysis were performed using the unweighted UniFrac distance algorithm and weighted UniFrac distance algorithm, and adonis analysis (permutational MANOVA) was used for otherness test. Then, based on the obtained community abundance data, a hypothesis test was performed using rigorous statistical methods to assess the significance level of species abundance differences between the microbial communities of the three groups of samples, and to obtain significantly different species between groups. LEfSe (linear discriminant analysis coupled with effect size analysis) performed linear discriminant analysis (LDA) on samples according to different grouping conditions based on taxonomic composition to find out the significantly different influences on the sample delineation of groups or species that had a significant differential impact on the sample delineation. The OTU abundance table was normalized by PICRUSt1. The effect of the number of copies of the 16S marker gene in the species genome was removed; then the COG corresponding to the OTU was obtained by the greengene corresponding to each OTU family information and KEGG Ortholog (KO) information for each OTU; and calculate the abundance of each COG and KO abundance. According to the information of COG database, the descriptive information of each COG and its functional information can be parsed from the eggNOG database to obtain the potential functional abundance spectrum; according to the information of KEGG database, the KO Pathway can be obtained, and the abundance of each potential functional category can be calculated according to the OTU abundance.

### Statistical analysis

The software mothur (version_1.30.2) was used for Alpha diversity analysis. Principal component analysis and principal co-ordinates analysis were statistically analysed and plotted using R (version 3.3.1). In NMDS analysis, Quantitative Insight Into Microbial Ecology 1 (QIIME, version_1.9.1) was applied to calculate the distance matrix of beta diversity, and then the R packages “vegan” and “mixOmics” were used for analysis and mapping. LEfSe (http://huttenhower.sph.harvard.edu/galaxy/roottool_id=lefse_upload) was used for multilevel species difference discriminant analysis; PICRUSt (version_1.1.0) software was used for functional prediction. All statistical calculations were performed in R 3.3.1. The Kruskal-Wallia H test was used to compare the differences in the measurement data between the three groups, and the Mann Whitney U test was used to compare the differences between two pairs. P-value < 0.05 was considered to be statistically significant, and the correction of the P-value is responsible for the false discovery rate (FDR).

## Results

### Basic clinical characteristics of patients and raw data management

A total of 24 EOCRC patients, 43 LOCRC patients and 31 healthy volunteers were recruited in this study. Their demographic characteristics are shown in **Table 1**. We collected 98 samples and obtained a total of 5,362,431 sequence fragments with a total length of 2,261,064,976 bps. The length of all samples ranged from 204 to 528 bp, with an average of 422 bp.

**Table 1:** Baseline information for three groups of patients.

Characteristics	EOCRC (n = 24)	LOCRC (n = 43)	NC (n = 31)
Gender (%)			
Male	17 (70.8%)	32 (74.4%)	22 (71.0%)
Female	7 (29.2%)	11 (25.6%)	9 (29.0%)
Age (years)			
Median	41	67	40
Range	26-49	55-79	21-46
BMI (kg/m^2^)			
Median	24.9	23.5	22.1
Range	17.9-31.6	16.2-31.6	17.3-30.1
History of Drinking (%)			
Yes	5 (20.8%)	9 (26.5%)	5 (16.1%)
No	19 (79.2%)	34 (73.5%)	26 (83.9%)
Tumor Site			
Colon	14 (58.3%)	25 (58.1%)	/
Rectum	10 (41.7%)	18 (41.9%)	/
TNM Staging (%)			
I	5 (20.8%)	9 (20.9%)	/
II	7 (29.2%)	14 (32.6%)	/
III	10 (41.7%)	17 (39.5%)	/
IV	2 (8.3%)	3 (7.0%)	/

EOCRC, early-onset colorectal cancer group; LOCRC, late-onset colorectal cancer group; NC, normal healthy young adults control group.

### Species assessment and species composition analysis

**Figure 1:**
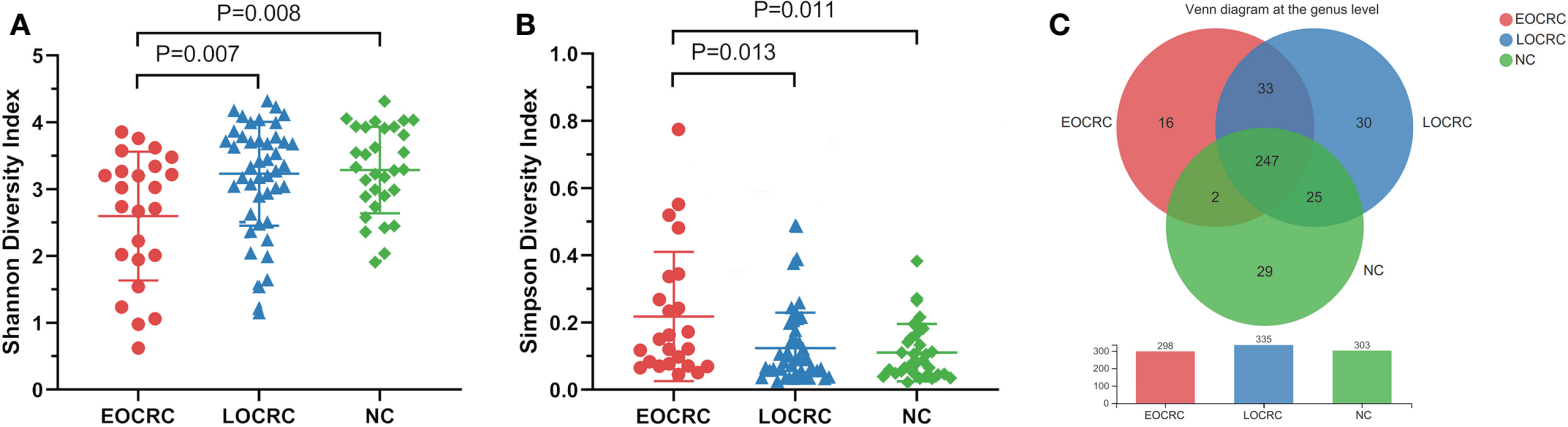
Alpha diversity analysis of gut microbiota in the three groups of patients. **A**: The comparison of Shannon index among the three groups. The Shannon index of the EOCRC group was significantly lower than the LOCRC group (P=0.007) and the NC group (P=0.008); **B**: The comparison of Simpson index among the three groups. The Simpson index of the EOCRC group was significantly higher than the LOCRC group (P=0.013) and the NC group (P=0.011); **C**: Venn diagram analysis of the three groups on the genus level. The three groups had 247 genera in common, with 16 unique genera in the EOCRC group, 30 unique genera in the LOCRC group and 29 unique genera in the NC group.

We performed OTU clustering on all valid sequences, and selected OTUs with the number of sequences greater than or equal to 5 in at least three samples and the sum of sequence numbers greater than or equal to 20, and finally obtained 714 OTUs, and the rank abundance curves are shown in **Figure S1A**. The Shannon curves of all samples can rapidly reach the plateau, indicating that the sequencing depth met the requirements. (**Figure S1B**) We performed alpha diversity analysis on the three groups and found that the Shannon diversity index of the EOCRC group was significantly lower than that of the LOCRC group (P=0.007) as well as that of the NC group (P=0.008). (**Figure 1A**) And the Simpson index of the EOCRC group was significantly lower than that of the LOCRC group (P=0.013) and that of NC group (P=0.011). (**Figure 1B**) The Venn diagram showed that at the genus level, the number of bacterial genera was higher in LOCRC group than EOCRC and NC groups, and the three groups shared 247 bacterial genera, with only 16 unique genera in EOCRC group. (**Figure 1C**)

### Beta-diversity analysis of gut microbiota

**Figure 2:**
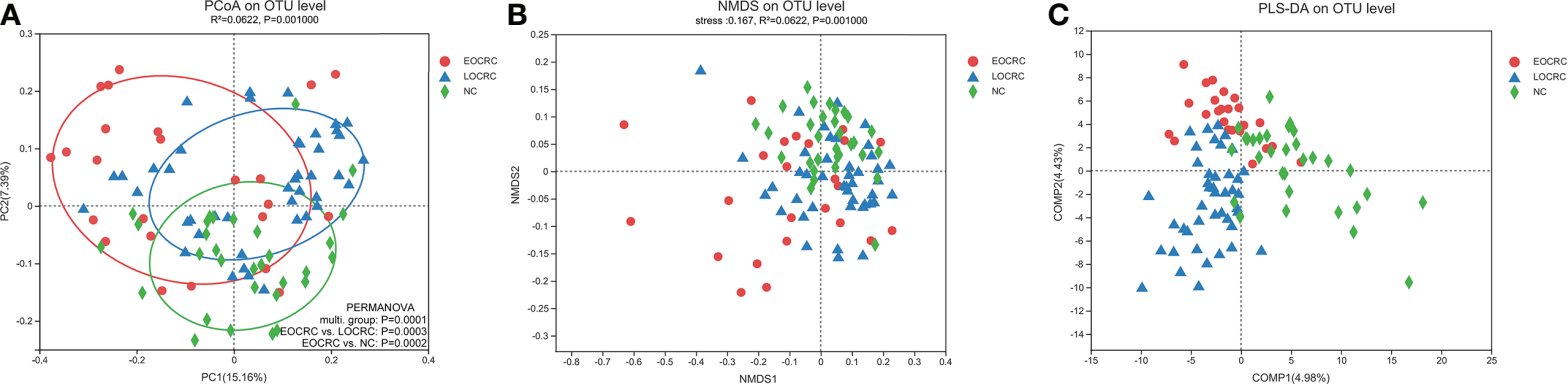
Beta diversity analysis of gut microbiota among the three groups. **(A)** Beta diversity calculated by PCoA of unweighted UniFrac distances and PERMANOVA. **(B)** The non-metric multidimensional scaling (NMDS) analysis results at the OUT level based on unweighted UniFrac distances. **(C)** Significant deviations were observed in the three sample groups at the OTU level.

We analyzed the difference of beta diversity among the three groups by PCoA, NMDS and PLS-DA. PCoA based on unweighted unifrac distance showed significant differences on the OTU level among the three groups (R²=0.0695, P=0.001), and adonis analysis showed significant differences between the EOCRC and LOCRC groups (P=0.0003) and between the EOCRC and NC groups (P=0.0002). (**Figure 2A**) PCoA based on weighted unifrac distances also showed significant differences among the three groups on OTU the level (R²= 0.0726, P=0.001). (**Figure S2A**) The results of the NMDS analysis on the OTU level were measured by the NMDS intensity index based on unweighted unifrac distance (stress=0.167, P=0.001, **Figure 2B**). The corresponding values based on weighted unifrac distance were as follows: OTU level (stress=0.136, P=0.001), genus level (stress=0.140, P=0.001) and phylum level (stress=0.073, P=0.001), as shown in **Figures S2B-D**. PLS-DA showed a clear separation of the three groups on the OTU level (**Figure 2C**). These data indicated that EOCRC harbored a peculiar microbiota.

### Gut microbiota dysbiosis in EOCRC

**Figure 3:**
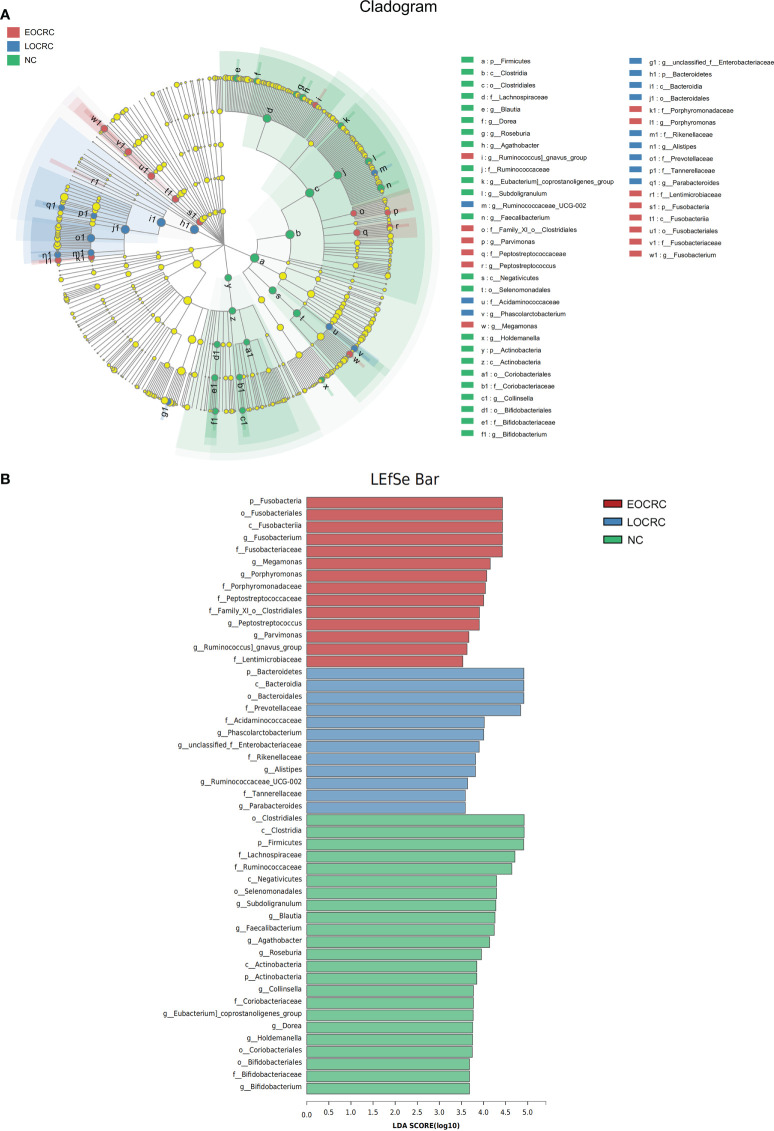
LEfSe algorithms were performed on the three groups. **(A)**: Cladogram measured from the LEfSe analysis. **(B)**: The LDA scores were obtained by linear regression analysis (LDA). The threshold for the linear discriminant analysis score was set at 3.5. The larger the LDA score, the greater the difference between the three groups.

We performed LEfSe to investigate the composition of fecal microbiota in the three groups and identify taxa that were differentially abundant in the EOCRC (linear discriminant analysis (LDA) score > 3.5, P-value < 0.05). There were 48 bacterial taxa whose relative abundances were significantly distinct among the three groups, with 14, 12 and 23 taxa increasing in the EOCRC, LOCRC and NC groups, respectively (**Figure 3A**). As show in **Figure 3B**, on the phylum (LDA score=4.4283, P<0.001), class (LDA score=4.4283, P<0.001), order (LDA score=4.4283, P<0.001), family (LDA score=4.4247, P<0.001), and genus (LDA score=4.4256, P<0.001) levels, *Fusobacteria* was mostly abundant, showed a strong relationship with EOCRC. And *Porphyromonas* was another abundant bacterium in EOCRC group on the family (LDA score=4.0416, P<0.001), and genus (LDA score=4.0714, P<0.001) levels. And *Bacteroidetes* (LDA score=4.9111, P=0.0011), *Bacteroidia* (LDA score=4.9111, P=0.0011), and *Bacteroidales* (LDA score= 4.9110, P=0.0011) were designated as the most powerful markers in LOCRC patients. However, in the NC group, significantly increased *Firmicutes* (LDA score=4.9069, P=0.0021), *Clostridia* (LDA score=4.9182, P=0.0022) and *Clostridiales* (LDA score=4.9182, P=0.0022) were considered as the most significant markers. We performed kruskal-wallis test on the abundance of bacteria in the three groups at different levels to verify the results of LEfSe analysis (**Table 2**). As shown in **Table 2**, in the EOCRC group, *Fusobacteria* was more abundant on the level of phylum (P<0.001), class (P<0.001), order (P<0.001), family (P<0.001) and genus (P<0.001); and *Porphyromonas* was more abundant on the genus level (P<0.001), but the proportion of *Porphyromonas* was low. And in the LOCRC group, the proportion of *Bacteroidetes* were significantly higher on the level of phylum (P=0.001113), class (P=0.001113), and order (P=0.001113). And *Prevotellaceae* was more abundant in the LOCRC group on the family level (P<0.001). In the NC group, *Clostridia* was more abundant on the class level (P=0.002217) and the order level (P=0.002217), and *Firmicutes* was enriched on the phylum level (P=0.002079). Another abundant bacterium in the NC group is *Actinobacteria*, which is more abundant at the phylum level and at the phylum level (all P-values=0.002197). These results were consistent with the LEfSe analysis. Therefore, we concluded that the specific bacteria in gut bacterial composition of the EOCRC, LOCRC and NC group were *Fusobacteria*, *Bacteroidetes*, and *Clostridia*, respectively.

**Table 2:** Taxa differentially represented in the gut microbiota of the three groups.

Taxa	EOCRC (%, n = 24)	LOCRC (%, n = 43)	NC (%, n = 31)	P value
**Phylum**				
Firmicutes	45.62 ± 23.68	43.45 ± 14.44	59.34 ± 20.08	0.002079
Bacteroidetes	23.49 ± 19.23	38.34 ± 21.31	21.96 ± 15.50	0.001113
Actinobacteria	1.783 ± 3.787	2.625 ± 4.786	3.580 ± 4.415	0.002197
Fusobacteria	4.565 ± 11.360	0.541 ± 1.324	0.207 ± 0.576	<0.001
**Class**				
Clostridia	31.88 ± 23.32	31.01 ± 15.72	47.82 ± 22.15	0.002217
Bacteroidia	23.49 ± 19.23	38.34 ± 21.31	21.96 ± 15.50	0.001113
Actinobacteria	1.783 ± 3.787	2.625 ± 4.786	3.580 ± 4.415	0.002197
Fusobacteriia	4.565 ± 11.360	0.541 ± 1.324	0.207 ± 0.576	<0.001
**Order**				
Clostridiales	31.88 ± 23.32	31.01 ± 15.72	47.82 ± 22.15	0.002217
Bacteroidales	23.49 ± 19.23	38.34 ± 21.31	21.96 ± 15.50	0.001113
Fusobacteriales	4.565 ± 11.360	0.541 ± 1.324	0.207 ± 0.576	<0.001
Bifidobacteriales	1.256 ± 3.490	1.633 ± 4.075	2.106 ± 3.430	0.002128
**Family**				
Lachnospiraceae	14.92 ± 11.94	14.37 ± 8.777	25.04 ± 15.67	0.005071
Ruminococcaceae	11.51 ± 14.85	13.41 ± 9.346	20.95 ± 20.95	0.007689
Bacteroidaceae	16.89 ± 17.17	16.18 ± 15.83	9.409 ± 10.83	0.1486
Prevotellaceae	2.913 ± 5.957	16.78 ± 16.78	9.307 ± 14.90	<0.001
Fusobacteriaceae	4.528 ± 11.360	0.535 ± 1.319	0.207 ± 0.576	<0.001
**Genus**				
Faecalibacterium	3.679 ± 7.249	4.369 ± 4.809	6.961 ± 7.190	0.005775
Blautia	3.213 ± 4.584	2.123 ± 1.773	5.716 ± 7.509	0.002130
Bacteroides	16.89 ± 17.17	16.18 ± 15.83	9.409 ± 10.83	0.1486
Fusobacterium	4.528 ± 11.360	0.535 ± 1.319	0.207 ± 0.576	<0.001
Porphyromonas	1.987 ± 5.338	0.708 ± 1.943	0.000304 ± 0.00114	<0.001

EOCRC, early-onset colorectal cancer group; LOCRC, late-onset colorectal cancer group; NC, normal healthy young adults control group.

### Functional analysis of fecal microbiota

**Figure 4:**
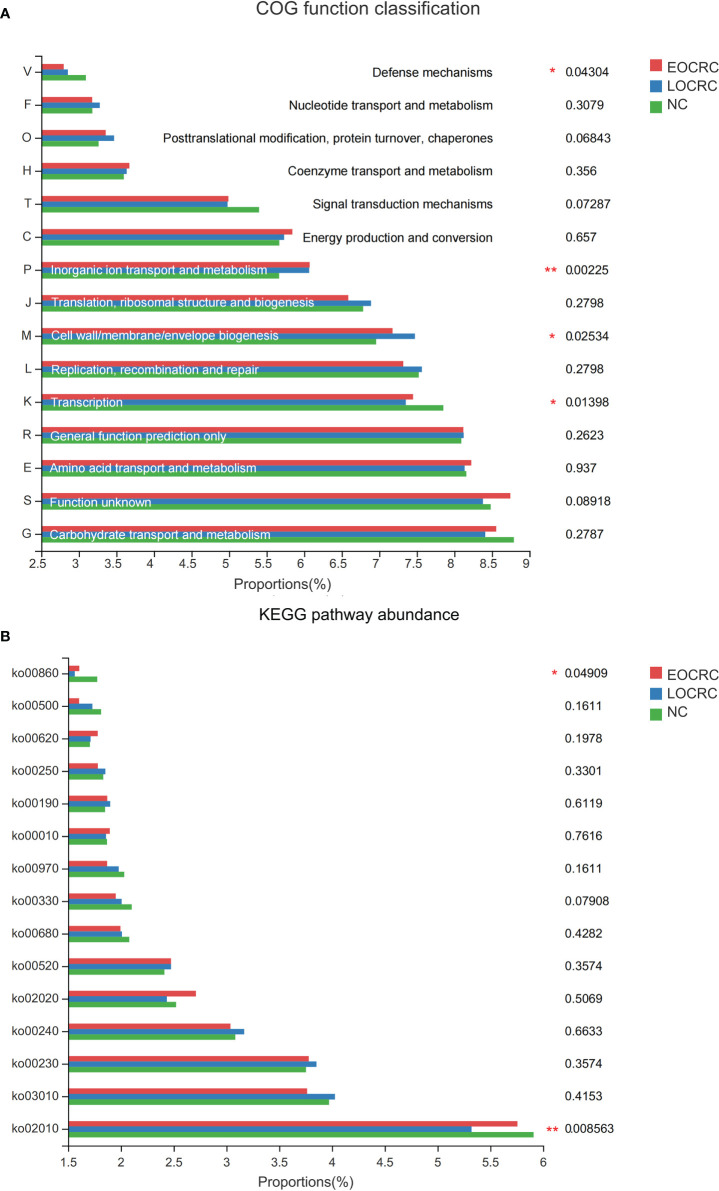
The function prediction of the three groups. **A**: The differences of Cluster of Ortholog Genes (COG) function. **B**: The abundance differences of the Kyoto Encyclopedia of Genes and Genomes **(KEGG)** pathway. 0.01 < corrected P-value ≤ 0.05 marked as *; 0.001 < corrected P-value ≤ 0.01 marked as **.

To study the functional and metabolic changes of the fecal microbial communities, we compared the measured sequences with the suggested database for the GOG and the KEGG module abundance from bacterial species. The COG potential functional annotation results showed that the EOCRC group as well as the LOCRC group were inferior in the following functions: transcription (P=0.01398) and defense mechanisms (P=0.04304). (**Figure 4A**, **Figure S3A**) Meanwhile, the three groups showed significant differences in the functions such as inorganic ion transport and metabolism (P=0.00225) and cell wall/membrane/envelope biogenesis (P=0.02534). (**Figure 4A**) Moreover, the KEGG modules involved in membrane transport (ko02010, P=0.00856) and porphyrin and chlorophyll metabolism (ko00860, P=0.04909) were overrepresented in the NC group compared with the EOCRC group and LOCRC group. (**Figure 4B**; **Figure S3B**)

## Discussion

The structure of the colorectal cancer population is gradually changing, and the rapidly increasing incidence of early-onset colorectal cancer requires vigilance (Collaborative et al., 2021; Sinicrope, 2022). The heterogeneity of clinical and molecular features of early-onset colorectal cancer is quite distinct, which means that it may be independent of traditional colorectal cancer (Silla et al., 2014; Fernandez-Rozadilla et al., 2021). As research progresses, the characteristics of the intestinal flora can be a major consideration in the etiology of many cancers (Murphy et al., 2019). Various studies have shown significant differences in the characteristics of gut microbiome across age, while the gut microbiome was considered to definite risk factor for colorectal cancer (O'Toole and Jeffery, 2015; Garrett, 2019; Wong and Yu, 2019). Therefore, we are more interested in clarifying the characteristics of gut microbiome in EOCRC. We selected patients with sporadic early-onset colorectal cancer from our center and recruited young healthy volunteers and late onset colorectal cancer patients with matched demographic characteristics. We initially delineated the gut flora of patients with sporadic colorectal cancer.

Prior studies have shown that imbalanced gut flora in CRC is usually manifested by a decrease in alpha diversity, however studies derived from Chinese populations suggest that the species diversity of gut microbiota of CRC patients is not different from that of healthy populations (Wang et al., 2012; Gagniere et al., 2016; Yachida et al., 2019). A metagenomic sequencing based study suggested that the faecal alpha diversity separation estimates of EOCRC patients were significantly lower than those of the LOCRC patients and healthy young volunteers (Kong et al., 2022). In this study, we found that EOCRC patients had significantly lower alpha diversity than the gut flora of LOCRC patients and healthy young volunteers. The abundance of gut microbiota in the EOCRC group was significantly lower than that in the LOCRC group and NC group, and the number of bacterial genera in the EOCRC group was the lowest of the three groups. The alpha diversity and richness of the gut microbiota are generally considered to be independent of age (Takagi et al., 2019). However, according to our findings, in colorectal cancer patients, the species diversity and abundance were significantly lower in young patients. Meanwhile, significant differences were found in the beta diversity of gut microbiota among the three groups for overall comparison as well as for pairwise comparisons. Combined with alpha diversity analysis and the microbiota variability analysis, it is reasonable to assume that there are some specificities in the gut microbiota of early-onset patients.

We compared the differences in abundant gut microbiota among the three groups. The proportion of *Bacteroides* in CRC patients, including EOCRC patients and in LOCRC patients was higher than that in NC patients (16.89 ± 17.17 *vs.* 16.18 ± 15.83 *vs.* 9.409 ± 10.83). But there was no obvious statistical difference among the three groups. Members of the genus *Bacteroides* account for a major fraction of the gut microbiome and colonize different parts of the colon (Kim et al., 2017). *Bacteroides fragilis* toxin can induce tumorigenesis through various pathways including IL17, signal transducer and activator of transcription 3 and nuclear factor-κB signaling in colonic epithelial cells (Chung et al., 2018). The *Bacteroidetes* were significantly enriched in the LOCRC group, and further analysis revealed that this part of the difference might be derived from a higher proportion of *Prevotellaceae*. Previous study has shown that *Prevotellaceae* was more abundant in CRC patients (Chen et al., 2012). However, there were only a small number of studies focusing on the association between *Prevotellaceae* and colorectal cancer. And exploring the role of *Prevotellaceae* in colorectal carcinogenesis may be a topic for future research. *Fusobacterium* is one of the definitive causative agents of CRC, and numerous studies have suggested that it can lead to colorectal carcinogenesis and progression (Mima et al., 2016; Yachida et al., 2019; Hong et al., 2021; Kong et al., 2021). In addition, *Fusobacterium* can promote chemoresistance in colorectal cancer by modulating autophagy, which can lead to poor prognosis in colorectal cancer patients (Yu et al., 2017). A previous study based on 16S rRNA gene sequencing suggested that *Fusobacterium* could serve as a differentially abundant genus marker for EOCRC, which could validate the results of the present study (Yang et al., 2021). Another study based on integrated metagenomic sequencing suggested that *Bacteroides vulgatus* and *Flavonifractor plautii* are unique taxon signatures for EOCRC, while *Fusobacterium* is a unique taxa signature for the LOCRC group (Kong et al., 2022). We suggest that differences in results are more likely to result from differences in sequencing methods and sample sources. Based on our study, *Fusobacterium* may play an important role in the gut microbiota of EOCRC patients, although it is present in lower proportions. Another genus enriched in the EOCRC group is *Porphyromonas*, and different species contained in it could promote colorectal carcinogenesis through butyrate-induced senescence or hematopoietic NLRP3 inflammasome (Okumura et al., 2021; Wang et al., 2021). In addition, we found a decrease in *Clostridia* in both the EOCRC group and the LOCRC group. *Clostridia* contains a variety of butyric acid-producing bacteria that can inhibit colorectal cancer development by modulating various signaling pathways and gut microbiota (Montalban-Arques et al., 2021; Stoeva et al., 2021; Zhou et al., 2022).

Through functional prediction, we found some changes in certain COG functions and KEGG pathways in each group. Compared with healthy volunteers, the EOCRC and LOCRC groups showed a significant decrease in some functions (such as transcription and defense mechanisms) and some KEGG pathways (such as membrane transport and porphyrin and chlorophyll metabolism). However, we speculated that these distinctions were more derived from the differences between CRC patients and healthy individuals. Although there was no clear mechanism to suggest the difference between gut microbiota and cellular function, we speculated that the gut microbiota can interact and regulate each other through certain specific signaling pathways with the host (Zmora et al., 2019). The functional changes in different groups necessarily produce tumorigenic or protective effects and may serve as targets for the next treatment of colorectal cancer.

Although our work has several novel findings, several limitations remain. The sample size of the control group (LOCRC group and NC group) of this study was adequate, but the sample size of the target population of our study needs to be larger. In addition, the male-to-female ratio of CRC patients in this study was slightly skewed, which may cause the findings of this study to be unrepresentative of the entire colorectal cancer population. Furthermore, metagenomic sequencing of the corresponding populations may give more convincing results.

In conclusion, our study suggests that patients with early-onset colorectal cancer have a unique gut microbial profile. Gut microbes could be another characteristic of early-onset colorectal cancer. We hope that this study will provide some insight into the use of gut microbes as biomarkers for predicting the risk of early-onset colorectal cancer and contribute to the prevention and treatment of early-onset colorectal cancer.

## Data availability statement

The datasets presented in this study can be found in online repositories. The names of the repository/repositories and accession number(s) can be found below: NCBI, PRJNA883949.

## Ethics statement

The studies involving human participants were reviewed and approved by the Ethics Review committee in the second affiliated hospital of Harbin Medical University. The patients/participants provided their written informed consent to participate in this study.

## Author contributions

HX, GW, and QT designed the project. JW, ZC, HH, ZY, YZ, ZH, and CW participated in patient selection and data collection. HX, YL, and YW carried out 16S sequencing, analyzed and interpreted the data. HX and JW preformed statistical analysis. HX, JW, and QT wrote the manuscript. All authors contributed to the article and approved the submitted version.

## Funding

This work was supported by the Applied Technology Research and Development Project of Heilongjiang Province (number GA19C003) and National Natural Science Foundation Youth Project (number 82103030).

## Conflict of interest

The authors declare that the research was conducted in the absence of any commercial or financial relationships that could be construed as a potential conflict of interest.

## Publisher’s note

All claims expressed in this article are solely those of the authors and do not necessarily represent those of their affiliated organizations, or those of the publisher, the editors and the reviewers. Any product that may be evaluated in this article, or claim that may be made by its manufacturer, is not guaranteed or endorsed by the publisher.

## Supplementary material

SUPPLEMENTARY FIGURE1 A. The Rank-Abundance curves of the three groups. B.The Shannon-Wiener curve.

SUPPLEMENTARY FIGURE 2 Beta diversity analysis based on unweighted unifiac distances. A. The PCoA analysis among the three groups on the level of OTU (R^2^=0.726, P-value=0.001); B,C,D. The NMDS analysis among the three groups on the level of OTU (stress=0.136, R^2^=0.0726, P-value=0.001), genus (stress=0.140, R^2^=0.0716, P-value=0.001) and phylum (stress=0.073, R^2^=0.1036, P-value=0.001).

SUPPLEMENTARY FIGURE 3 Pairwise comparisons of the functional predictions for the three groups. A. The differences of Cluster of Ortholog Genes (COG) function. B. The abundance differences of the Kyoto Encyclopedia of Genes and Genomes (KEGG) pathway. 0.01 < corrected P-value ≥ 0.05 marked as *; 0.001 < corrected P-value ≤ 0.01 marked as **.
