# Supplementary figures and images for: Gut microbiota display alternative profiles in patients with early-onset colorectal cancer
Source: Front Cell Infect Microbiol. 2022 Oct 27;12:1036946. doi: 10.3389/fcimb.2022.1036946 (PMC9648186; doi:10.3389/fcimb.2022.1036946)

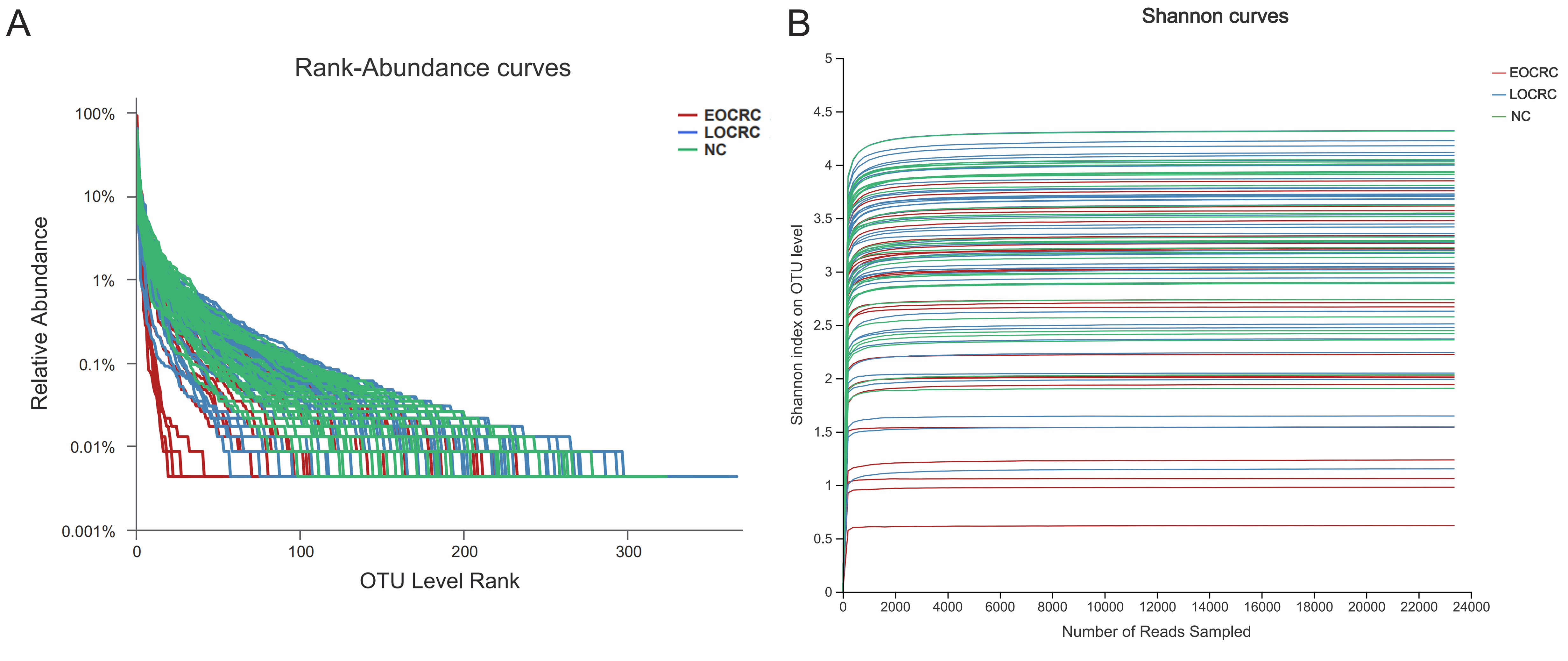

Supplement: SUPPLEMENTARY FIGURE1 — A. The Rank-Abundance curves of the three groups. B.The Shannon-Wiener curve. [file Image_1.jpeg]

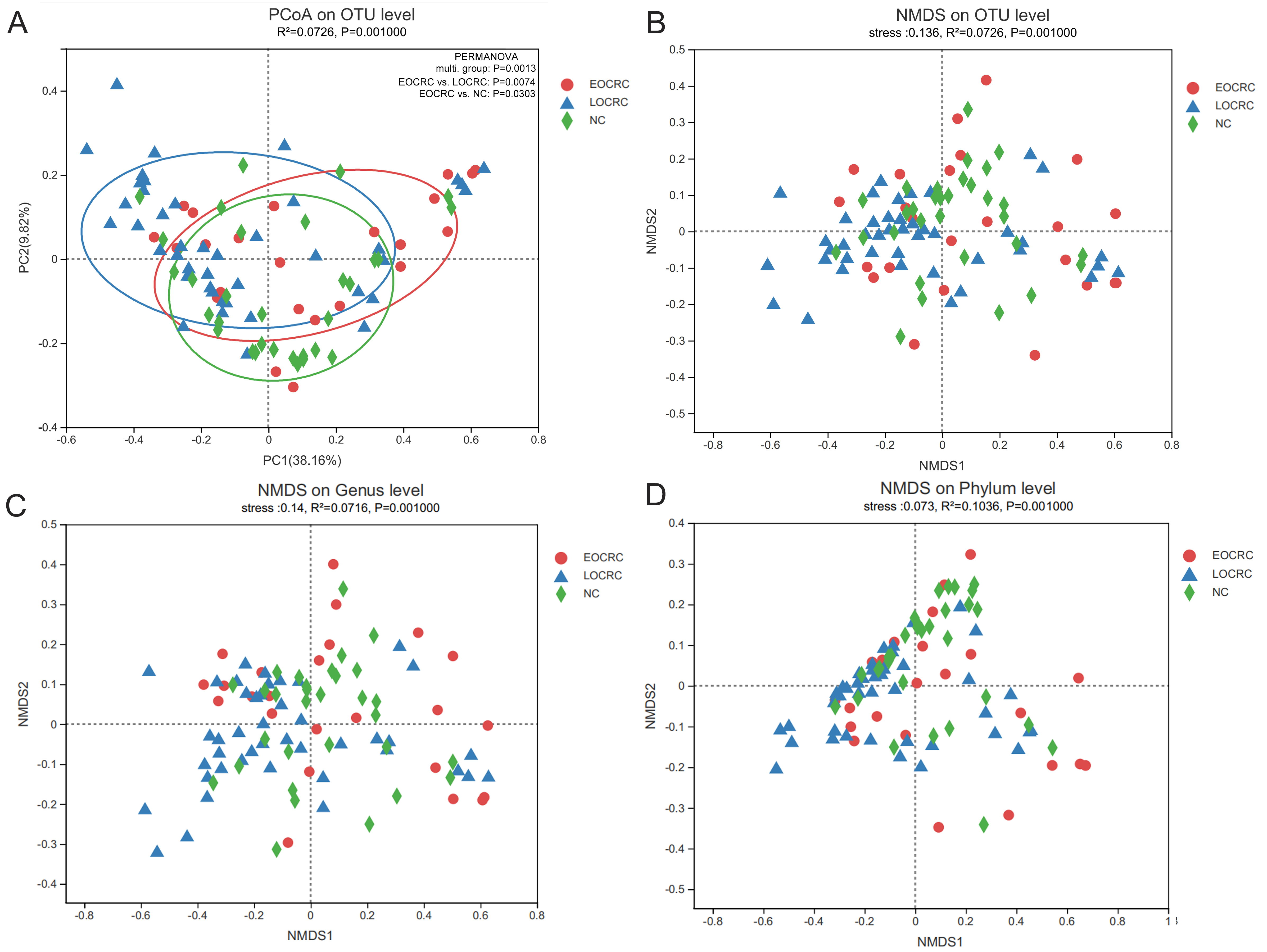

Supplement: SUPPLEMENTARY FIGURE 2 — Beta diversity analysis based on unweighted unifiac distances. A. The PCoA analysis among the three groups on the level of OTU (R2=0.726, P-value=0.001); B,C,D. The NMDS analysis among the three groups on the level of OTU (stress=0.136, R2=0.0726, P-value=0.001), genus (stress=0.140, R2=0.0716, P-value=0.001) and phylum (stress=0.073, R2=0.1036, P-value=0.001). [file Image_2.jpeg]

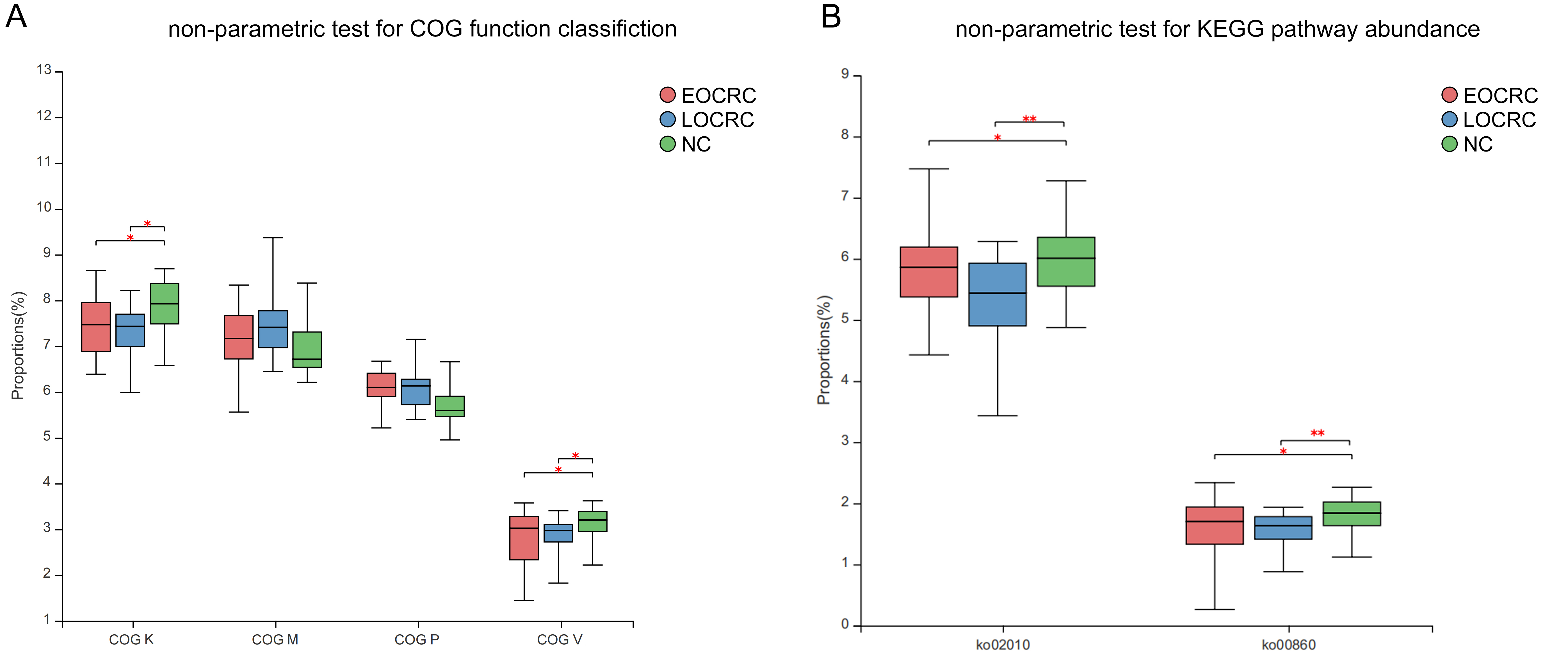

Supplement: SUPPLEMENTARY FIGURE 3 — Pairwise comparisons of the functional predictions for the three groups. A. The differences of Cluster of Ortholog Genes (COG) function. B. The abundance differences of the Kyoto Encyclopedia of Genes and Genomes (KEGG) pathway. 0.01 < corrected P-value ≥ 0.05 marked as *; 0.001 < corrected P-value ≤ 0.01 marked as **. [file Image_3.jpeg]
